# 2-Methyl-4-(2-methyl­benzamido)­benzoic acid

**DOI:** 10.1107/S160053681101110X

**Published:** 2011-04-07

**Authors:** Fei-Fei He, Ya-Bin Shi, Song Xia, Hai-Bo Wang

**Affiliations:** a College of Food Science and Light Industry, Nanjing University of Technology, Xinmofan Road No. 5 Nanjing, Nanjing 210009, People’s Republic of China; b College of Science, Nanjing University of Technology, Xinmofan Road No. 5 Nanjing, Nanjing 210009, People’s Republic of China

## Abstract

In the crystal structure of the title compound, C_16_H_15_NO_3_, inter­molecular N—H⋯O hydrogen bonds link the mol­ecules into chains parallel to the *b* axis and pairs of inter­molecular O—H⋯O hydrogen bonds between inversion-related carb­oxy­lic acid groups link the mol­ecules into dimers. The dihedral angle between the two benzene rings is 82.4 (2)°.

## Related literature

For the use of the title compound as an inter­mediate in the preparation of pharmaceutically active benzazepine compounds that have vasopressin antagonistic activity, see: Yasuhiro *et al.* (2007 ▶). For the preparation of the title compound, see: Yasuhiro *et al.* (2000 ▶). For bond-length data, see: Allen *et al.* (1987 ▶).
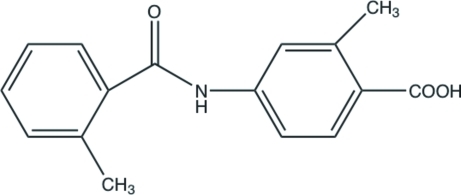


## Experimental

### 

#### Crystal data

C_16_H_15_NO_3_
*M* *_r_* = 269.29
Monoclinic, 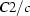

*a* = 23.318 (9) Å
*b* = 10.230 (2) Å
*c* = 13.901 (3) Å
β = 125.50 (3)°
*V* = 2699.7 (16) Å^3^
*Z* = 8
Mo *K*α radiation
μ = 0.09 mm^−1^
*T* = 293 K
0.20 × 0.10 × 0.10 mm

#### Data collection

Enraf–Nonius CAD-4 diffractometer
Absorption correction: ψ scan (North *et al.*, 1968 ▶) *T*_min_ = 0.982, *T*_max_ = 0.991
4968 measured reflections
2493 independent reflections
1641 reflections with *I* > 2σ(*I*)
*R*_int_ = 0.034
3 standard reflections every 120 min  intensity decay: 1%

#### Refinement

*R*[*F* ^2^ > 2σ(*F* ^2^)] = 0.047
*wR*(*F* ^2^) = 0.138
*S* = 1.00
2493 reflections
184 parameters
H-atom parameters constrained
Δρ_max_ = 0.19 e Å^−3^
Δρ_min_ = −0.18 e Å^−3^

### 

Data collection: *CAD-4 EXPRESS* (Enraf–Nonius, 1989 ▶); cell refinement: *CAD-4 EXPRESS*; data reduction: *XCAD4* (Harms & Wocadlo, 1995 ▶); program(s) used to solve structure: *SHELXS97* (Sheldrick, 2008 ▶); program(s) used to refine structure: *SHELXL97* (Sheldrick, 2008 ▶); molecular graphics: *SHELXTL* (Sheldrick, 2008 ▶); software used to prepare material for publication: *PLATON* (Spek, 2009) ▶.

## Supplementary Material

Crystal structure: contains datablocks global, I. DOI: 10.1107/S160053681101110X/pk2306sup1.cif

Structure factors: contains datablocks I. DOI: 10.1107/S160053681101110X/pk2306Isup2.hkl

Additional supplementary materials:  crystallographic information; 3D view; checkCIF report

## Figures and Tables

**Table 1:** Hydrogen-bond geometry (Å, °)

*D*—H⋯*A*	*D*—H	H⋯*A*	*D*⋯*A*	*D*—H⋯*A*
N—H0*A*⋯O1^i^	0.86	2.23	3.076 (3)	169
O2—H2*A*⋯O3^ii^	0.82	1.82	2.636 (4)	174

Symmetry codes: (i) 
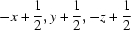
; (ii) 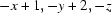.

**Fig. 1.**
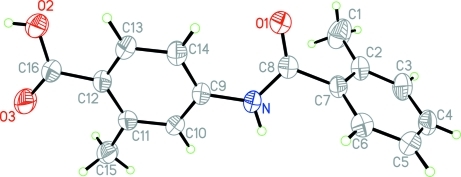
The molecular structure of the title compound, with displacement ellipsoids drawn at the 50% probability level.

**Fig. 2.**
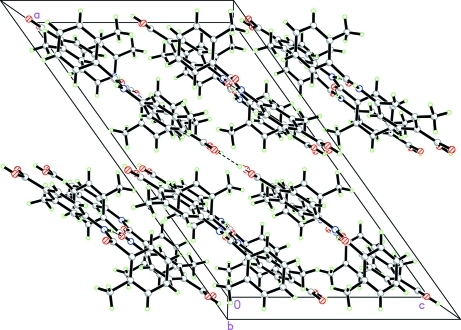
A packing diagram of 2-methyl-4-(2-methylbenzamido)benzoic acid viewed down the b axis. Hydrogen bonds are shown as dashed lines.

## supplementary crystallographic information

### Comment

The title compound, 2-methyl-4-(2-methylbenzamido)benzoic acid, or salts thereof are useful as intermediates for preparing pharmaceutically active benzazepine compounds that have vasopressin antagonistic activity. (Yasuhiro *et al.* 2007).

In the molecule of 2-methyl-4-(2-methylbenzamido)benzoic acid (Fig. 1), the bond lengths (Allen *et al.*, 1987) and angles are within normal ranges. Intermolecular N-H···O hydrogen bonds link the molecules parallel to the *b* axis and pairs of intermolecular N-H···O hydrogen bonds link the molecules parallel to the *b* axis and pairs of intermolecular O-H···O hydrogen bonds between inversion related (x,y,z & 1-x,2-y,-z) carboxylic acid groups link the molecules into dimers. The dihedral angle between the two benzene rings is 82.39 (19) ° (Fig. 2).

### Experimental

The title compound, 2-methyl-4-(2-methylbenzamido)benzoic acid was prepared by the literature method (Yasuhiro *et al.* 2000). Recrystallization of the of the crude crystalline product gave a yield of 81%.   Crystals suitable for X-ray analysis were obtained by slow evaporation of a solution in methanol.

### Refinement

H atoms were positioned geometrically, with N-H = 0.86 Å (for NH) and O-H =0.82 Å (for OH) and C-H = 0.93, 0.98 and 0.96 Å for aromatic, methine and methyl H, respectively. They were constrained to ride on their parent atoms, with U_iso_(H) values set to either 1.2U_eq_ or 1.5U_eq_ (RCH_3_, OH) of the attached atom.

### Crystal data

**Table:**

C_16_H_15_NO_3_	*F*(000) = 1136
*M**_r_* = 269.29	*D*_x_ = 1.325 Mg m^−^^3^
Monoclinic, *C*2/*c*	Melting point: 497 K
Hall symbol: -C 2yc	Mo *K*α radiation, λ = 0.71073 Å
*a* = 23.318 (9) Å	Cell parameters from 25 reflections
*b* = 10.230 (2) Å	θ = 9–13°
*c* = 13.901 (3) Å	µ = 0.09 mm^−^^1^
β = 125.50 (3)°	*T* = 293 K
*V* = 2699.7 (16) Å^3^	Block, colourless
*Z* = 8	0.20 × 0.10 × 0.10 mm

### Data collection

**Table:**

Enraf–Nonius CAD-4 diffractometer	1641 reflections with *I* > 2σ(*I*)
Radiation source: fine-focus sealed tube	*R*_int_ = 0.034
graphite	θ_max_ = 25.4°, θ_min_ = 2.2°
ω/2θ scans	*h* = −28→28
Absorption correction: ψ scan (North *et al.*, 1968)	*k* = 0→12
*T*_min_ = 0.982, *T*_max_ = 0.991	*l* = −16→16
4968 measured reflections	3 standard reflections every 120 min
2493 independent reflections	intensity decay: 1%

### Refinement

**Table:**

Refinement on *F*^2^	Secondary atom site location: difference Fourier map
Least-squares matrix: full	Hydrogen site location: inferred from neighbouring sites
*R*[*F*^2^ > 2σ(*F*^2^)] = 0.047	H-atom parameters constrained
*wR*(*F*^2^) = 0.138	*w* = 1/[σ^2^(*F*_o_^2^) + (0.075*P*)^2^] where *P* = (*F*_o_^2^ + 2*F*_c_^2^)/3
*S* = 1.00	(Δ/σ)_max_ < 0.001
2493 reflections	Δρ_max_ = 0.19 e Å^−^^3^
184 parameters	Δρ_min_ = −0.18 e Å^−^^3^
0 restraints	Extinction correction: *SHELXL*, Fc^*^=kFc[1+0.001xFc^2^λ^3^/sin(2θ)]^-1/4^
Primary atom site location: structure-invariant direct methods	Extinction coefficient: 0.0023 (5)

### Special details

**Table:**

Geometry. All esds (except the esd in the dihedral angle between two l.s. planes) are estimated using the full covariance matrix. The cell esds are taken into account individually in the estimation of esds in distances, angles and torsion angles; correlations between esds in cell parameters are only used when they are defined by crystal symmetry. An approximate (isotropic) treatment of cell esds is used for estimating esds involving l.s. planes.
Refinement. Refinement of F^2^ against ALL reflections. The weighted R-factor wR and goodness of fit S are based on F^2^, conventional R-factors R are based on F, with F set to zero for negative F^2^. The threshold expression of F^2^ > 2σ(F^2^) is used only for calculating R-factors(gt) etc. and is not relevant to the choice of reflections for refinement. R-factors based on F^2^ are statistically about twice as large as those based on F, and R- factors based on ALL data will be even larger.

### Fractional atomic coordinates and isotropic or equivalent isotropic displacement parameters (Å^2^)

**Table:**

	*x*	*y*	*z*	*U*_iso_*/*U*_eq_	
N	0.27879 (10)	1.06074 (17)	0.23814 (17)	0.0431 (5)	
H0A	0.2725	1.1380	0.2547	0.052*	
O1	0.25277 (9)	0.84642 (15)	0.23884 (17)	0.0573 (5)	
C1	0.09320 (14)	0.8941 (3)	0.0956 (2)	0.0698 (8)	
H1A	0.0432	0.8865	0.0559	0.105*	
H1B	0.1141	0.8086	0.1159	0.105*	
H1C	0.1026	0.9364	0.0445	0.105*	
O2	0.45652 (10)	0.90523 (16)	0.04728 (17)	0.0639 (6)	
H2A	0.4798	0.9040	0.0202	0.096*	
C2	0.12408 (12)	0.9737 (2)	0.2061 (2)	0.0432 (6)	
O3	0.46865 (10)	1.11880 (17)	0.03858 (18)	0.0662 (6)	
C3	0.08066 (13)	1.0191 (2)	0.2371 (2)	0.0507 (7)	
H3A	0.0328	1.0002	0.1884	0.061*	
C4	0.10675 (14)	1.0914 (2)	0.3382 (2)	0.0496 (7)	
H4A	0.0764	1.1214	0.3562	0.060*	
C5	0.17705 (14)	1.1195 (2)	0.4121 (2)	0.0490 (6)	
H5A	0.1949	1.1656	0.4818	0.059*	
C6	0.22118 (12)	1.0787 (2)	0.3823 (2)	0.0440 (6)	
H6A	0.2688	1.0997	0.4312	0.053*	
C7	0.19543 (12)	1.0070 (2)	0.2804 (2)	0.0372 (5)	
C8	0.24493 (12)	0.9618 (2)	0.2510 (2)	0.0400 (5)	
C9	0.32340 (11)	1.0500 (2)	0.2002 (2)	0.0387 (5)	
C10	0.32865 (11)	1.1578 (2)	0.1449 (2)	0.0420 (6)	
H10A	0.3043	1.2335	0.1373	0.050*	
C11	0.36888 (12)	1.1568 (2)	0.1007 (2)	0.0409 (6)	
C12	0.40456 (11)	1.0408 (2)	0.1123 (2)	0.0404 (6)	
C13	0.39995 (12)	0.9348 (2)	0.1700 (2)	0.0458 (6)	
H13A	0.4244	0.8589	0.1787	0.055*	
C14	0.36050 (12)	0.9378 (2)	0.2148 (2)	0.0459 (6)	
H14A	0.3589	0.8657	0.2540	0.055*	
C15	0.37002 (14)	1.2772 (2)	0.0391 (2)	0.0555 (7)	
H15A	0.3369	1.3400	0.0312	0.083*	
H15B	0.3574	1.2538	−0.0378	0.083*	
H15C	0.4164	1.3144	0.0848	0.083*	
C16	0.44573 (12)	1.0256 (2)	0.0627 (2)	0.0452 (6)	

### Atomic displacement parameters (Å^2^)

**Table:**

	*U*^11^	*U*^22^	*U*^33^	*U*^12^	*U*^13^	*U*^23^
N	0.0528 (12)	0.0369 (10)	0.0640 (13)	−0.0009 (9)	0.0480 (11)	−0.0035 (9)
O1	0.0742 (12)	0.0373 (9)	0.0957 (14)	−0.0002 (8)	0.0695 (12)	−0.0015 (9)
C1	0.0601 (18)	0.098 (2)	0.0525 (17)	−0.0085 (16)	0.0333 (15)	−0.0222 (16)
O2	0.0834 (13)	0.0489 (10)	0.1041 (15)	−0.0071 (9)	0.0799 (13)	−0.0164 (10)
C2	0.0471 (14)	0.0498 (14)	0.0434 (14)	−0.0013 (11)	0.0324 (12)	0.0026 (11)
O3	0.0883 (14)	0.0514 (11)	0.1102 (16)	−0.0040 (9)	0.0870 (14)	−0.0038 (10)
C3	0.0415 (14)	0.0655 (16)	0.0562 (16)	0.0015 (12)	0.0348 (13)	0.0037 (13)
C4	0.0609 (16)	0.0479 (14)	0.0690 (17)	0.0030 (12)	0.0542 (15)	0.0028 (13)
C5	0.0685 (17)	0.0427 (13)	0.0559 (16)	−0.0053 (12)	0.0476 (15)	−0.0077 (11)
C6	0.0479 (14)	0.0427 (13)	0.0512 (15)	−0.0059 (11)	0.0344 (13)	−0.0044 (11)
C7	0.0451 (13)	0.0355 (11)	0.0451 (13)	0.0019 (10)	0.0342 (12)	0.0042 (10)
C8	0.0438 (13)	0.0395 (13)	0.0471 (13)	−0.0004 (10)	0.0324 (12)	−0.0003 (10)
C9	0.0408 (12)	0.0395 (12)	0.0493 (14)	−0.0042 (10)	0.0339 (12)	−0.0061 (10)
C10	0.0490 (14)	0.0334 (12)	0.0606 (15)	−0.0011 (10)	0.0416 (13)	−0.0061 (11)
C11	0.0466 (13)	0.0371 (12)	0.0522 (14)	−0.0085 (10)	0.0362 (12)	−0.0096 (11)
C12	0.0412 (13)	0.0423 (13)	0.0511 (14)	−0.0057 (10)	0.0344 (12)	−0.0081 (11)
C13	0.0477 (14)	0.0400 (12)	0.0665 (17)	0.0055 (10)	0.0426 (14)	−0.0009 (11)
C14	0.0498 (14)	0.0413 (13)	0.0655 (16)	0.0021 (11)	0.0442 (14)	0.0040 (12)
C15	0.0724 (18)	0.0418 (14)	0.0803 (19)	0.0006 (12)	0.0603 (16)	0.0031 (13)
C16	0.0472 (14)	0.0442 (13)	0.0595 (16)	−0.0033 (11)	0.0398 (13)	−0.0101 (12)

### Geometric parameters (Å, °)

**Table:**

N—C8	1.357 (3)	C5—H5A	0.9300
N—C9	1.417 (3)	C6—C7	1.386 (3)
N—H0A	0.8600	C6—H6A	0.9300
O1—C8	1.221 (3)	C7—C8	1.503 (3)
C1—C2	1.501 (3)	C9—C14	1.380 (3)
C1—H1A	0.9600	C9—C10	1.389 (3)
C1—H1B	0.9600	C10—C11	1.389 (3)
C1—H1C	0.9600	C10—H10A	0.9300
O2—C16	1.300 (3)	C11—C12	1.404 (3)
O2—H2A	0.8200	C11—C15	1.509 (3)
C2—C3	1.391 (3)	C12—C13	1.388 (3)
C2—C7	1.398 (3)	C12—C16	1.481 (3)
O3—C16	1.230 (3)	C13—C14	1.379 (3)
C3—C4	1.378 (4)	C13—H13A	0.9300
C3—H3A	0.9300	C14—H14A	0.9300
C4—C5	1.367 (4)	C15—H15A	0.9600
C4—H4A	0.9300	C15—H15B	0.9600
C5—C6	1.378 (3)	C15—H15C	0.9600
			
C8—N—C9	126.85 (18)	O1—C8—C7	122.33 (19)
C8—N—H0A	116.6	N—C8—C7	113.80 (18)
C9—N—H0A	116.6	C14—C9—C10	119.50 (19)
C2—C1—H1A	109.5	C14—C9—N	122.87 (19)
C2—C1—H1B	109.5	C10—C9—N	117.63 (18)
H1A—C1—H1B	109.5	C11—C10—C9	122.6 (2)
C2—C1—H1C	109.5	C11—C10—H10A	118.7
H1A—C1—H1C	109.5	C9—C10—H10A	118.7
H1B—C1—H1C	109.5	C10—C11—C12	117.56 (19)
C16—O2—H2A	109.5	C10—C11—C15	118.97 (19)
C3—C2—C7	117.3 (2)	C12—C11—C15	123.43 (18)
C3—C2—C1	119.7 (2)	C13—C12—C11	119.16 (18)
C7—C2—C1	123.1 (2)	C13—C12—C16	118.4 (2)
C4—C3—C2	121.7 (2)	C11—C12—C16	122.4 (2)
C4—C3—H3A	119.1	C14—C13—C12	122.6 (2)
C2—C3—H3A	119.1	C14—C13—H13A	118.7
C5—C4—C3	120.4 (2)	C12—C13—H13A	118.7
C5—C4—H4A	119.8	C13—C14—C9	118.5 (2)
C3—C4—H4A	119.8	C13—C14—H14A	120.7
C4—C5—C6	119.3 (2)	C9—C14—H14A	120.7
C4—C5—H5A	120.4	C11—C15—H15A	109.5
C6—C5—H5A	120.4	C11—C15—H15B	109.5
C5—C6—C7	120.8 (2)	H15A—C15—H15B	109.5
C5—C6—H6A	119.6	C11—C15—H15C	109.5
C7—C6—H6A	119.6	H15A—C15—H15C	109.5
C6—C7—C2	120.50 (19)	H15B—C15—H15C	109.5
C6—C7—C8	119.7 (2)	O3—C16—O2	122.23 (19)
C2—C7—C8	119.8 (2)	O3—C16—C12	123.2 (2)
O1—C8—N	123.87 (19)	O2—C16—C12	114.6 (2)
			
C7—C2—C3—C4	−1.5 (3)	C8—N—C9—C10	152.9 (2)
C1—C2—C3—C4	179.4 (2)	C14—C9—C10—C11	1.5 (3)
C2—C3—C4—C5	−0.7 (4)	N—C9—C10—C11	−177.8 (2)
C3—C4—C5—C6	2.4 (4)	C9—C10—C11—C12	0.7 (3)
C4—C5—C6—C7	−1.8 (3)	C9—C10—C11—C15	178.4 (2)
C5—C6—C7—C2	−0.5 (3)	C10—C11—C12—C13	−2.0 (3)
C5—C6—C7—C8	−179.0 (2)	C15—C11—C12—C13	−179.6 (2)
C3—C2—C7—C6	2.1 (3)	C10—C11—C12—C16	176.5 (2)
C1—C2—C7—C6	−178.8 (2)	C15—C11—C12—C16	−1.2 (4)
C3—C2—C7—C8	−179.5 (2)	C11—C12—C13—C14	1.2 (4)
C1—C2—C7—C8	−0.4 (3)	C16—C12—C13—C14	−177.3 (2)
C9—N—C8—O1	5.4 (4)	C12—C13—C14—C9	0.9 (4)
C9—N—C8—C7	−173.8 (2)	C10—C9—C14—C13	−2.2 (4)
C6—C7—C8—O1	121.0 (3)	N—C9—C14—C13	177.0 (2)
C2—C7—C8—O1	−57.5 (3)	C13—C12—C16—O3	−160.2 (2)
C6—C7—C8—N	−59.8 (3)	C11—C12—C16—O3	21.4 (4)
C2—C7—C8—N	121.7 (2)	C13—C12—C16—O2	19.2 (3)
C8—N—C9—C14	−26.4 (4)	C11—C12—C16—O2	−159.2 (2)

### Hydrogen-bond geometry (Å, °)

**Table:**

*D*—H···*A*	*D*—H	H···*A*	*D*···*A*	*D*—H···*A*
N—H0A···O1^i^	0.86	2.23	3.076 (3)	169
O2—H2A···O3^ii^	0.82	1.82	2.636 (4)	174

Symmetry codes: (i) −*x*+1/2, *y*+1/2, −*z*+1/2; (ii) −*x*+1, −*y*+2, −*z*.
